# Supplementary material for: Natural variation in a type-A response regulator confers maize chilling tolerance
Source: Nat Commun. 2021 Aug 5;12:4713. doi: 10.1038/s41467-021-25001-y (PMC8342596; doi:10.1038/s41467-021-25001-y)
Supplement: Supplementary file 1 — Supplementary Information file [file 41467_2021_25001_MOESM1_ESM.pdf]

Natural variation in a type-A response regulator gene confers maize chilling tolerance

Zeng *et al.*

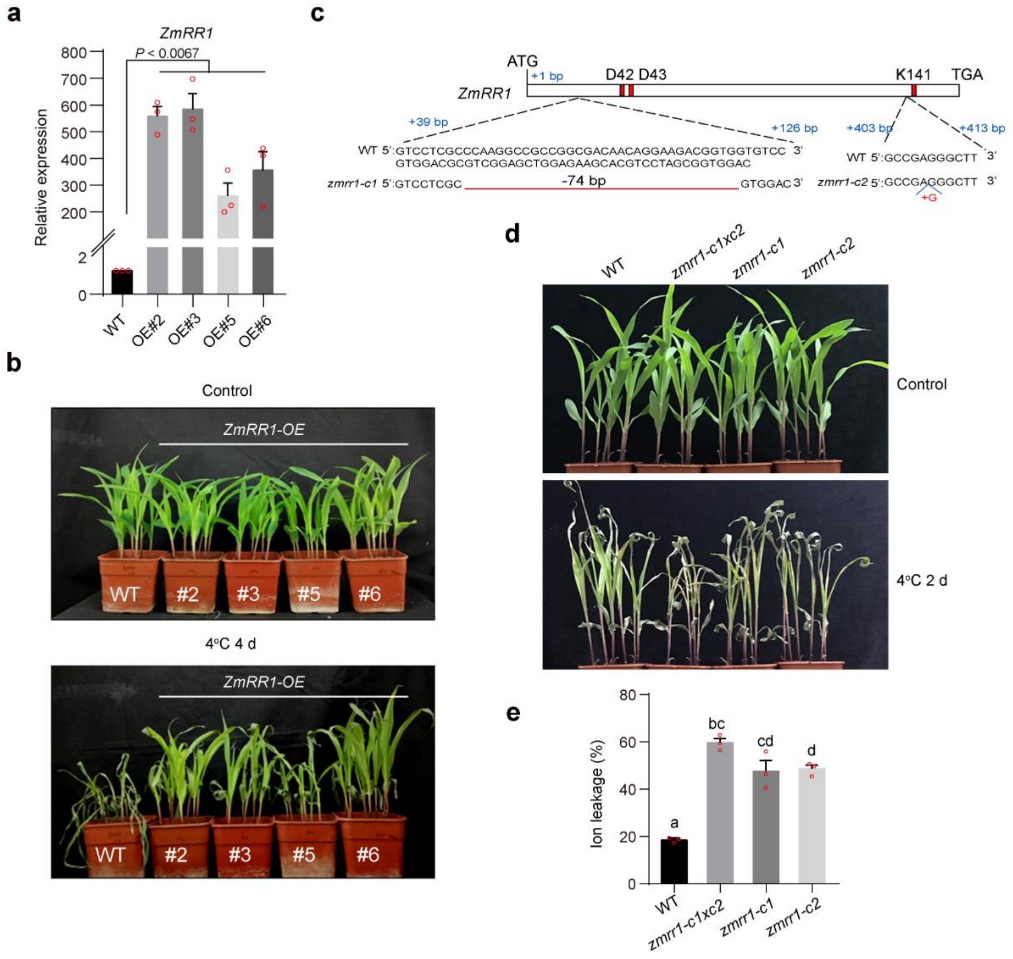

Supplementary Figure 1. Characterization of *ZmRR1*-overexpression transgenic plants and *zmrr1-crispr* mutants.

a Relative expression levels of *ZmRR1* in wild-type and *ZmRR1*-overexpression seedlings (*OE#2*, *OE#3*, *OE#5*, *OE#6*). Total RNAs were extracted from 10-day-old seedlings and subjected to qRT-PCR analysis. Relative expression levels in wild-type plants were set to 1.0. Data are the mean values ± SD (n = 3; two-sided t-test). A representative experiment from 3 independent experiments is shown. **b** Chilling phenotype of *ZmRR1*-overexpression transgenic seedlings (*OE#2*, *OE#3*, *OE#5*, *OE#6*) under cold conditions. 12-day-old seedlings grown at 25°C were incubated at 4°C for 4 d. Representative images were taken after 2 days of recovery. A representative experiment from 3 independent experiments is shown. **c** Schematics of mutations in the coding region of *ZmRR1*. CRISPR/Cas9 technology was used to generate two mutant alleles, *zmrr1-c1* and *zmrr1-c2*. **d**–**e** Chilling phenotype (**d**) and ion leakage (**e**) of wild-type, *zmrr1-c1*, *zmrr1-c2* and *zmrr1-c1*×*zmrr1-c2* (F1 plants obtained by crossing *zmrr1-c1* with *zmrr1-c2*) under cold conditions. 14-day-old seedlings grown at 25°C were incubated at 4°C for 2 d. Representative images were taken after 2 days of recovery. In **e**, each bar represents the mean ± SD (n = 3). Different letters represent significant difference at $P < 0.05$ (one-way ANOVA). A representative experiment from 3 independent experiments is shown. Source data underlying Supplementary Figure 1a and 1e are provided as a Source Data file.

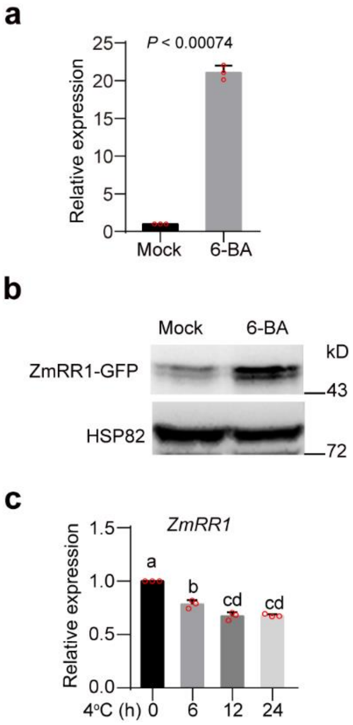

Supplementary Figure 2. The transcription and protein levels of ZmRR1 in response to exogenous cytokinin.

a Relative expression level of *ZmRR1* in 10-day-old wild-type seedlings treated with 10 μM 6-BA or DMSO for 15 h. Relative expression level in wild-type plants without treatment was set to 1.0. Data are the mean values ± SD (n = 3; two-sided Student's t-test). **b** The protein level of ZmRR1-GFP in protoplasts expressing ZmRR1-GFP by exogenous 10 μM 6-BA and incubated in darkness for 15 h. Total proteins were extracted and detected with anti-GFP antibody. HSP82 was used as a control. **c,** *ZmRR1* gene expression under cold stress. Relative expression in wild-type plants at 0 h was set to 1.0. Data are means ± SD (n = 3; $P < 0.05$, one-way ANOVA and Duncan's multiple comparison test). In **a-c**, a representative experiment from 3 independent experiments is shown. Source data are provided as a Source Data file.

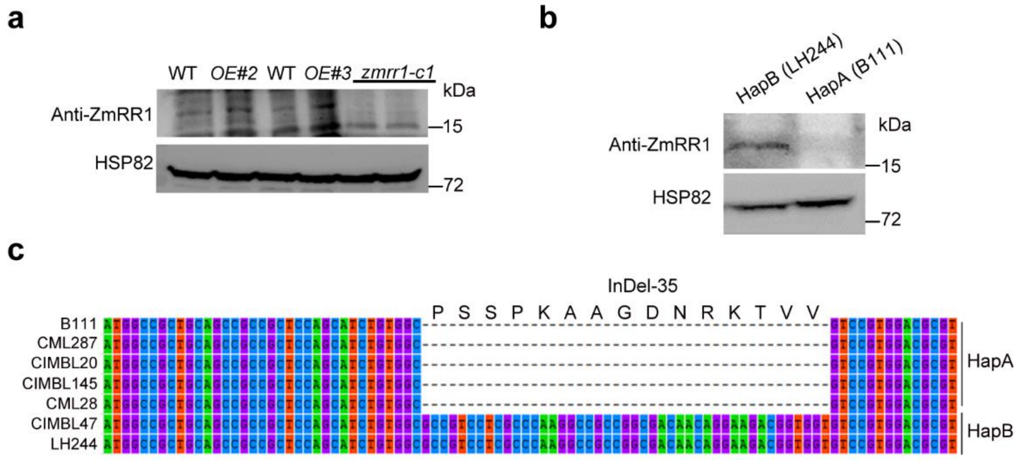

Supplementary Figure 3. Anti-ZmRR1 antibody can specifically detect ZmRR1 (ZmRR1^{HapB}) protein in maize.

a Immunoblot analysis of ZmRR1 protein in total proteins extracted from 10-day-old wild-type, *ZmRR1-OE* and *zmrr1-c1* mutant seedlings, respectively. **b** Immunoblot analysis of ZmRR1 protein in total proteins extracted from 10-day-old HapA (B111) and HapB (LH244) inbred lines. The 15 amino acid peptide (SSPKAAGDNRKTVVS) at the N-terminal of ZmRR1 was selected for anti-ZmRR1 antibody preparation. The 1-14 aa of this peptide was deleted in HapA. Therefore, this anti-ZmRR1 antibody can specifically detect ZmRR1^{HapB} but not ZmRR1^{HapA} protein. **c** DNA sequence alignment of Indel-35 in *ZmRR1* coding region with HapA and HapB representative maize inbred lines using MEGA 7.0 software. Source data underlying Supplementary Figure 3a and 3b are provided as a Source Data file.

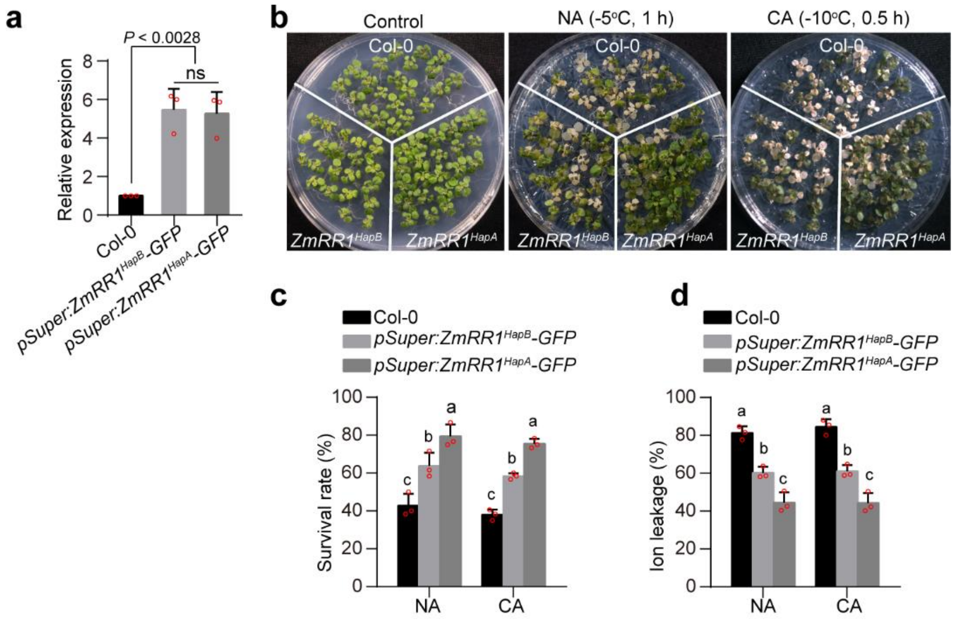

Supplementary Figure 4. Freezing tolerance of transgenic *Arabidopsis* plants overexpressing ZmRR1^{HapA} and ZmRR1^{HapB}.

a *ZmRR1* expression in transgenic *Arabidopsis* plants overexpressing ZmRR1^{HapA} and ZmRR1^{HapB}. **b**–**d** Freezing phenotypes (**b**), survival rates (**c**), and ion leakage (**d**) of transgenic *Arabidopsis* plants overexpressing ZmRR1^{HapA} and ZmRR1^{HapB} under non-acclimated (NA) and cold-acclimated (CA) conditions. 14-day-old seedlings grown on half-strength MS plates at 22°C were treated at -5°C for 1 h (NA) or were treated at -10°C for 1 h after pretreated at 4°C for 3 days (CA). In **a**, each bar represents the mean ± SD (n = 3; two-sided Student's t-test). In **c** and **d**, each bar represents the mean ± SD of three independent experiments. Different letters represent significant difference at P < 0.05 (one-way ANOVA). Source data underlying Supplementary Figure 4a, 4c, and 4d are provided as a Source Data file.

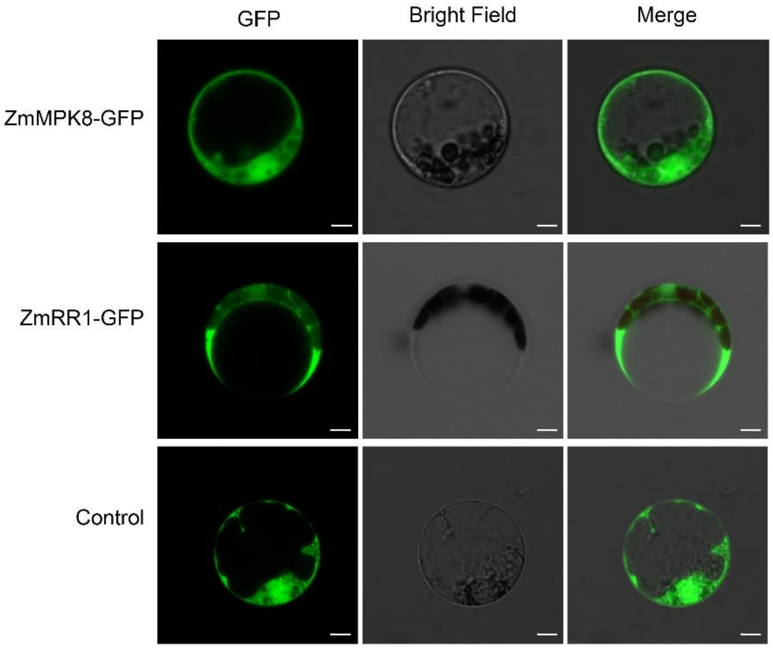

Supplementary Figure 5. Subcellular localization of ZmMPK8 and ZmRR1. *Super:ZmMPK8-GFP* and *Super:ZmRR1-GFP* were transformed into maize mesophyll protoplasts, respectively. *Super:1300-GFP* was used as a control. GFP signals were visualized by confocal microscope. Scale bars, 5 μm. A representative experiment from 3 independent experiments is shown.

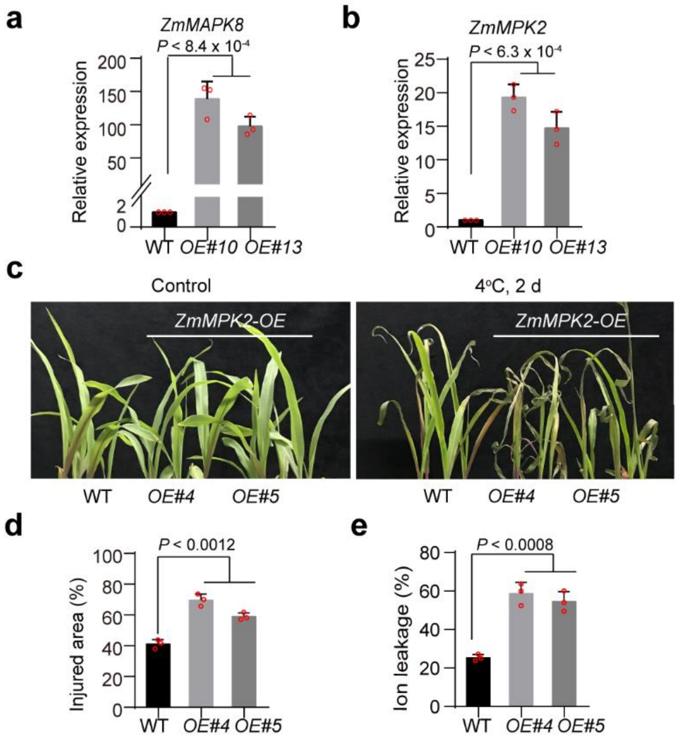

Supplementary Figure 6. Characterization of *ZmMPK8-OE* and *ZmMPK2-OE* plants.

a–**b** Relative expression levels of *ZmMPK8* (**a**) and *ZmMPK2* (**b**) in *ZmMPK8-OE* (**a**) and *ZmMPK2-OE* (**b**) transgenic lines. Total RNAs were extracted from 10-day-old seedlings and subjected to qRT-PCR analysis. Relative expression in wild-type plants was set to 1.0. Data are the mean values ± SD (n =3). A representative experiment from 3 independent experiments is shown. **c**–**e** Chilling phenotypes (**c**), ion leakage (**d**), and injured area (**e**) of *ZmMPK2-OE* transgenic plants (*OE#4*, *OE#5*) under cold conditions. 14-day-old seedlings grown at 25°C were incubated at 4°C for 2 d. In **d** and **e**, each bar represents the mean ± SD of three independent experiments. In **a**, **b**, **d** and **e**, the statistical significance was determined by a two-sided t-test. Source data underlying Supplementary Figure 6a, 6b, 6d, and 6e are provided as a Source Data file.

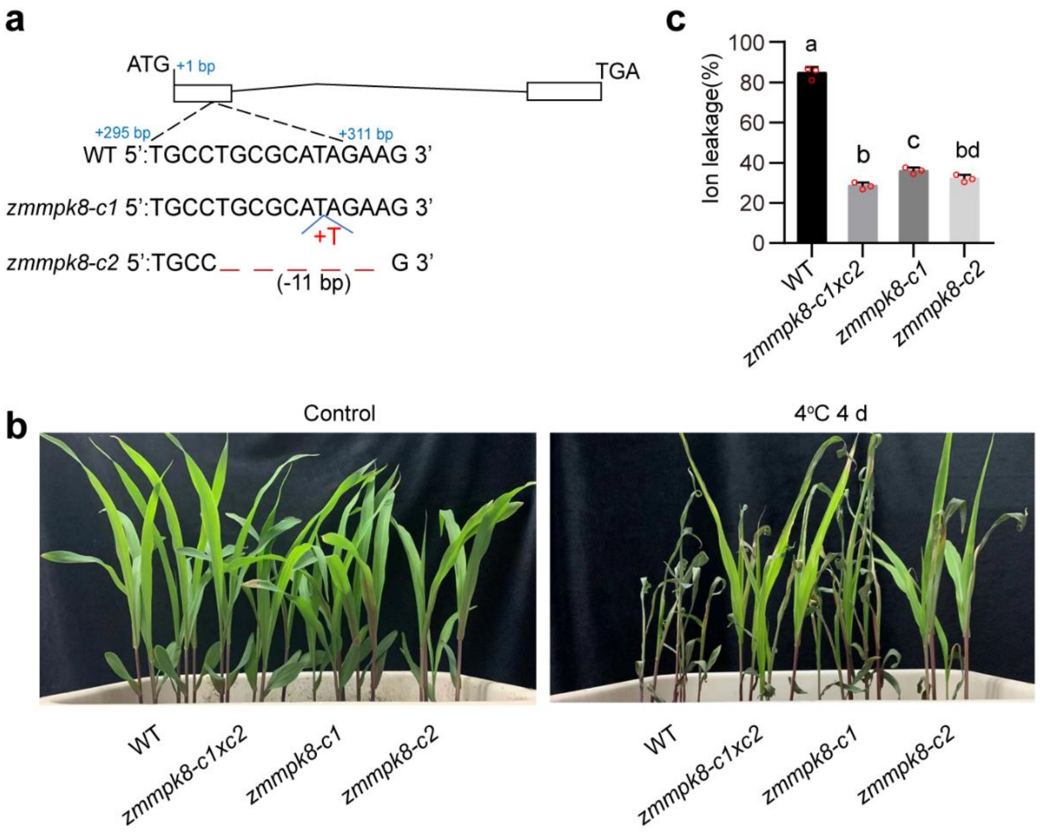

Supplementary Figure 7. Allelism test of *zmmpk8* mutants.

a Schematics of mutations in the coding region of *ZmMPK8*. CRISPR/Cas9 technology was used to generate two alleles of *zmmpk8* mutants, *zmmpk8-c1* and *zmmpk8-c2*. **b**–**c** Chilling phenotype (**b**), and ion leakage (**c**) of wild-type, *zmmpk8-c1*, *zmmpk8-c2* and *zmmpk8-c1* × *zmmpk8-c2* (F1 plants obtained by crossing *zmmpk8-c1* with *zmmpk8-c2*) under cold conditions. In **c**, each bar represents the mean ± SD ($n = 3$). Different letters represent significant difference at $P < 0.05$ (one-way ANOVA). A representative experiment from 3 independent experiments is shown. Source data underlying Supplementary Figure 7c are provided as a Source Data file.

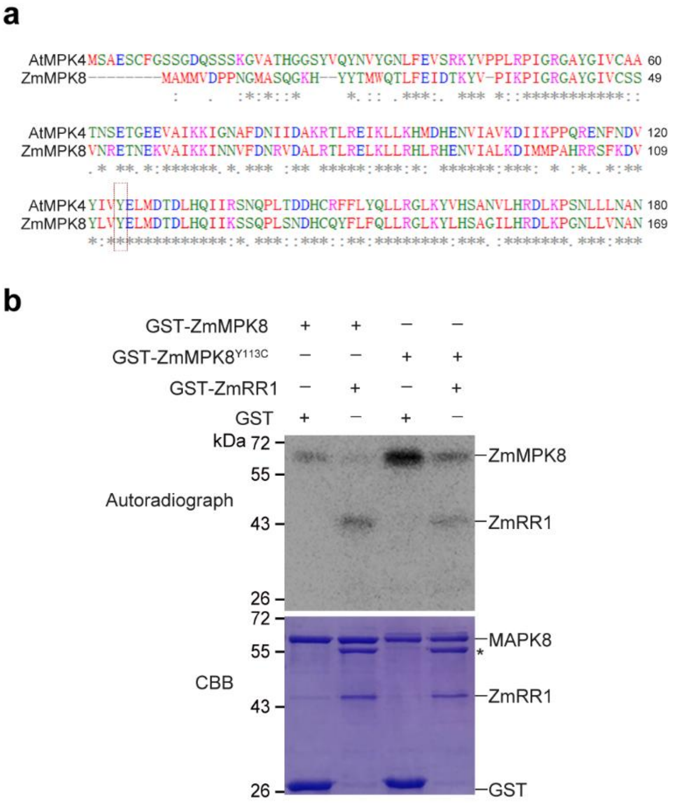

Supplementary Figure 8. ZmMPK8 and ZmMPK8^{Y113C} phosphorylate ZmRR1^{HapB} (ZmRR1) *in vitro*.

a Alignment analysis of amino acid sequences of ZmMPK8 and AtMPK4. **b** Purified recombinant GST, GST-ZmMPK8 and GST-ZmMPK8^{Y113C} proteins were separated by 10% SDS-PAGE following incubation with GST-ZmRR1 in protein kinase buffer containing [γ-^{32}P] ATP. Phosphorylated ZmRR1 was detected by autoradiography following gel electrophoresis (top panel). Recombinant proteins were detected by Coomassie brilliant blue (CBB) staining (bottom panel). Asterisk represents non-specific bands. In **b**, a representative experiment from 3 independent experiments is shown. Source data underlying Supplementary Figure 8b are provided as a Source Data file.

a

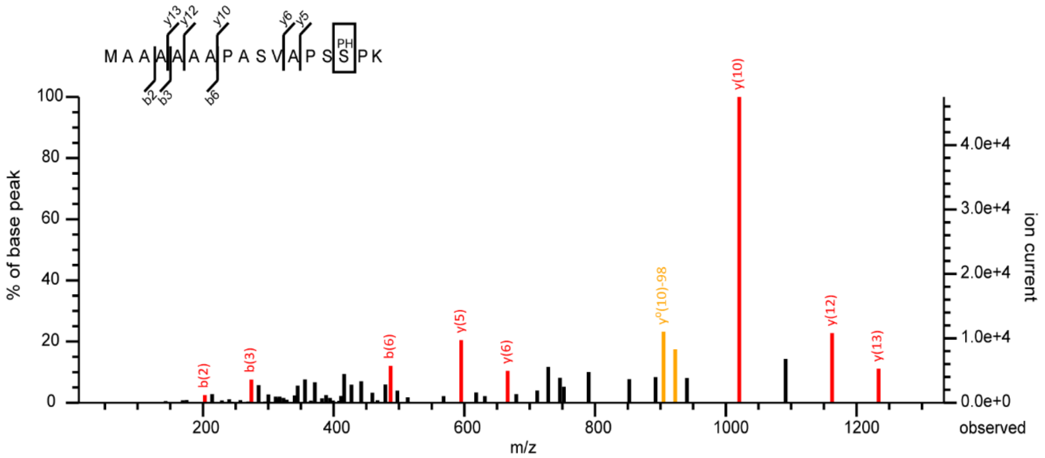

b

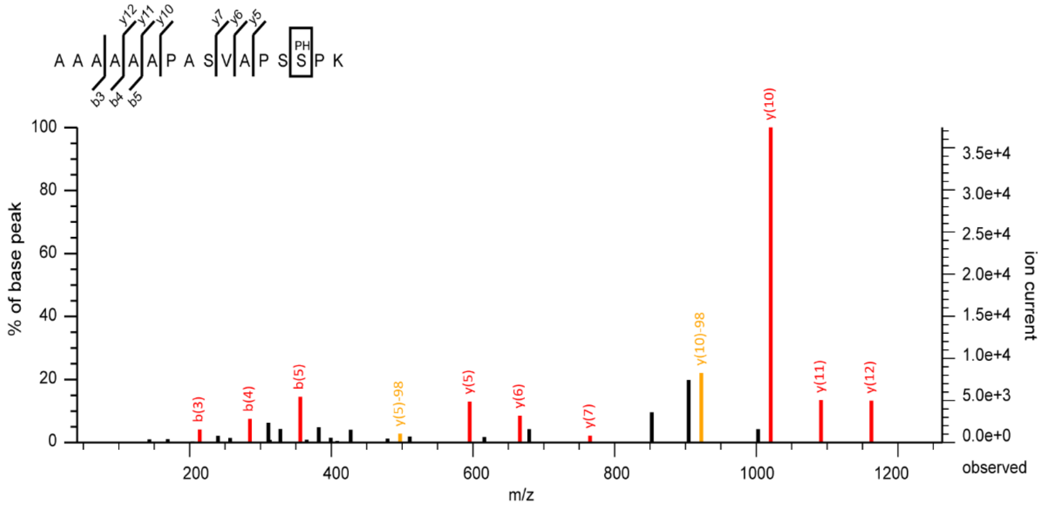

Supplementary Figure 9. Identification of phospho-peptides of ZmRR1 by LC-MS/MS.

a LC-MS/MS spectrum of phosphor-peptide MAAAAAAPASVAPS$_{P}$SPK (Ser15) *in vitro*. Purified recombinant GST-ZmRR1, and His-ZmMPK8 proteins were incubated in protein kinase buffer with or without ATP at 30°C for 30 min. **b** LC-MS/MS spectrum of phosphor-peptide MAAAAAAPASVAPS$_{P}$SPK (Ser15) *in vivo*. Maize protoplasts expressing ZmRR1-MYC were incubated in darkness for 15 h. Total proteins were extracted and immunoprecipitated with MYC beads and then performed mass spectrometry *in vivo*. Ser15 was identified as a phosphorylation site *in vivo*.

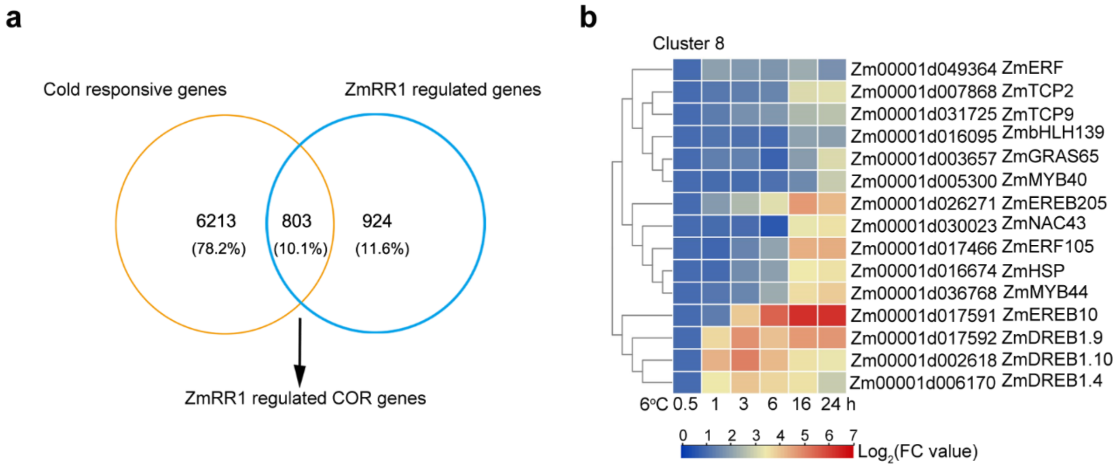

Supplementary Figure 10. Transcriptome profiling of *ZmRR1*.

a The Venn diagram showing ZmRR1-regulated *COR* genes. **b** Heat map showing the ZmRR1-regulated transcription factors in cluster 8 under cold stress using RNAseq data published[43]. Colors represent log2-fold change comparing relative expression at 6°C for 0.5 h.

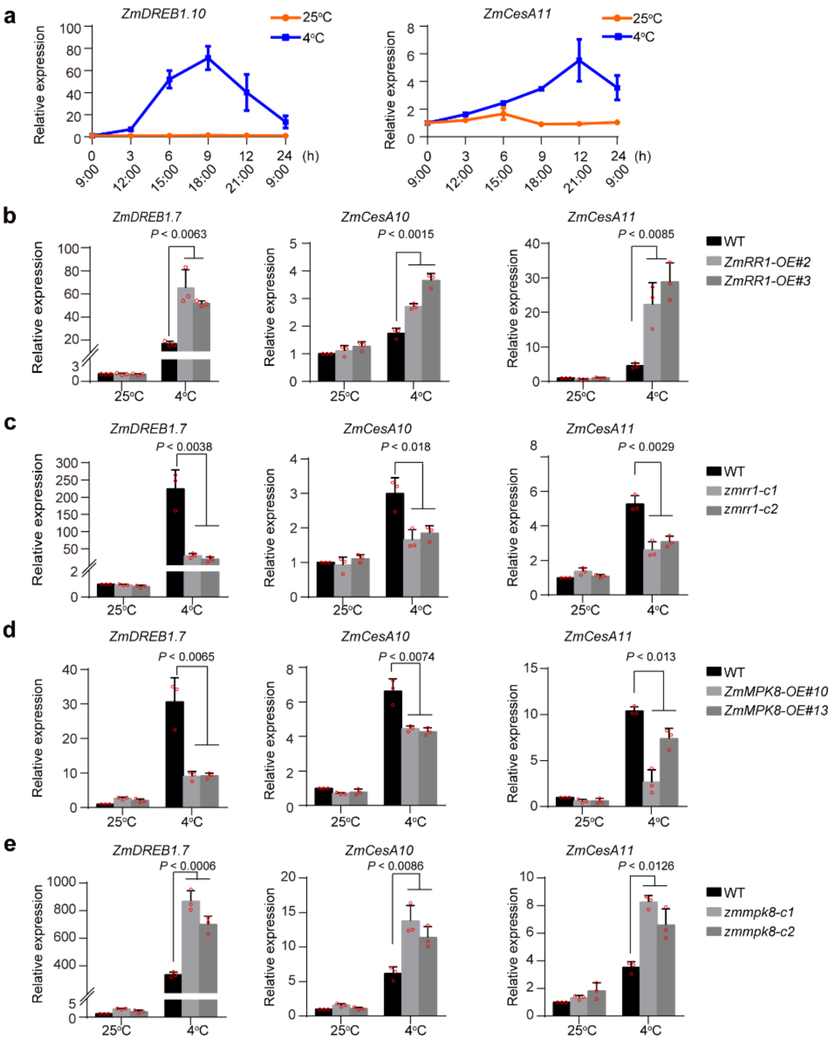

Supplementary Figure 11. ZmRR1 regulates chilling tolerance by modulating the expression of *ZmDREB1.7* and *ZmCesAs*.

a Relative expression levels of *ZmDREB1.10* and *ZmCesA11* for time-course cold treatment. **b**–**d** Relative expression levels of *ZmDREB1.7*, *ZmCesA10*, and *ZmCesA11* in *ZmRR1-OE* (**b**) and *zmrr1* plants (**c**) *ZmMPK8-OE* (**d**) and *zmmpk8* plants (**e**) under cold stress. Total RNA was extracted from 10-day-old seedlings following incubation at 25°C or 4°C for the indicated times and used for qRT-PCR analysis. Relative expression levels in wild-type plants at 25°C were set to 1.0. In **a**–**e**, data are the mean values ± SD (n =3). The statistical significance was determined by a two-sided t-test. A representative experiment from 3 independent experiments is shown. Source data are provided as a Source Data file.

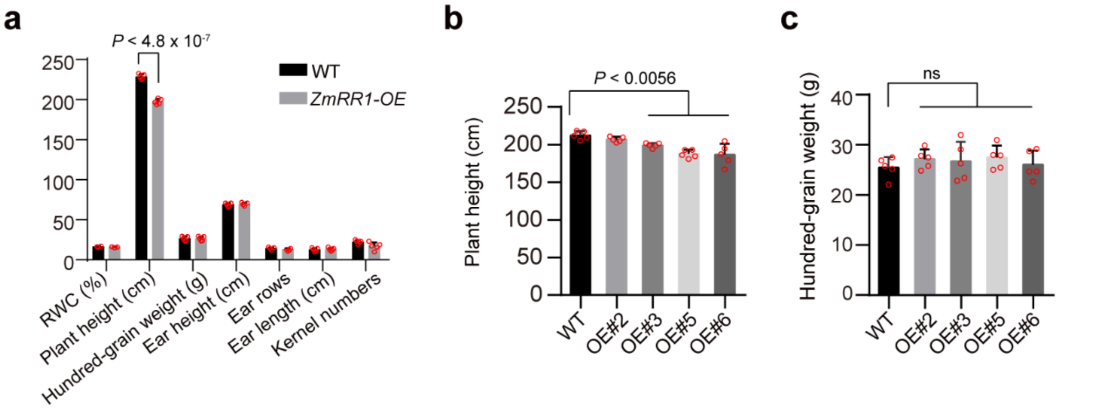

Supplementary Figure 12. Yield-related traits of *ZmRR1*-overexpressing lines.
a Comparison of relative water content (%), ear height, plant height, hundred-grain weight, ear rows, ear length and kernel numbers between wild-type and *ZmRR1* overexpression lines under optimal feeding conditions. **b**–**c** The plant height (**b**) and hundred-grain weight (**c**) of wild-type and *ZmRR1-OE* seedlings. Maize was planted in Gongzhuling, China (North latitude 43.31°, East longitude 124.49°) in 2016. In **a**–**c**, data are the mean values ± SD (n = 5 of independent experiments). The statistical significance was determined by a two-sided t-test. Source data are provided as a Source Data file.

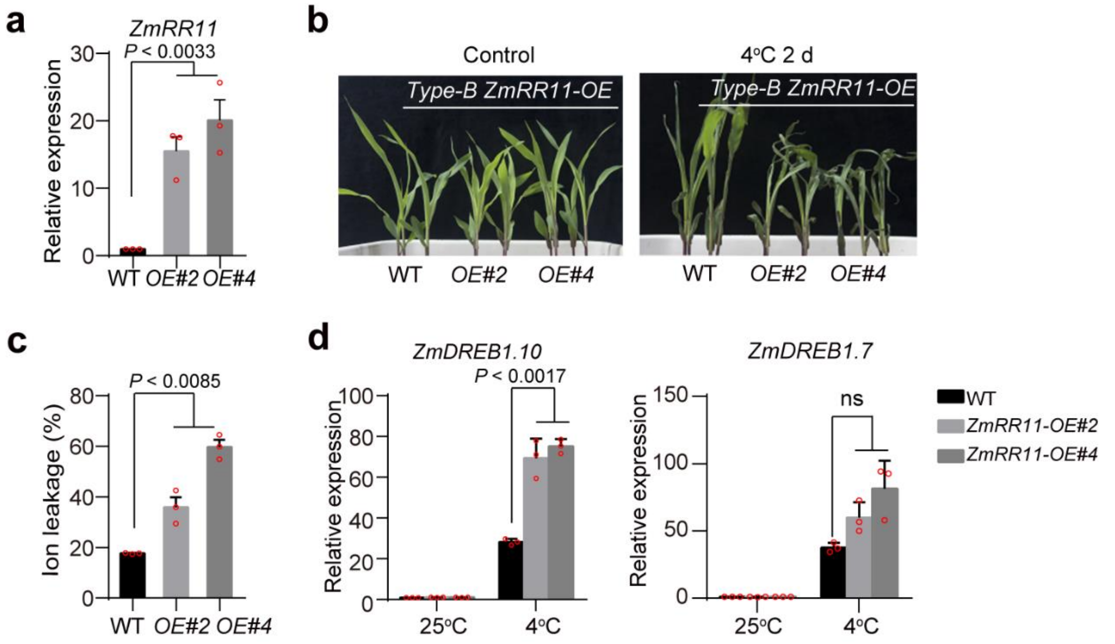

Supplementary Figure 13. Overexpression of type-B *ZmRR11* significantly reduces chilling tolerance.

a–c Relative expression level of *ZmRR11* (**a**), chilling phenotypes (**b**), and ion leakage (**b**) of type-B *ZmRR11*-overexpression transgenic plants (*OE#2*, *OE#4*) under cold treatment. **d** Relative expression levels of *ZmDREB1.7* and *ZmDREB1.10* in wild-type and type-B *ZmRR11-OE* transgenic plants after cold treatment. In **a** and **d**, data are the mean values ± SD ($n = 3$). A representative experiment from 3 independent experiments is shown. In **c**, each bar represents the mean ± SD of three independent experiments. The statistical significance was determined by a two-sided *t*-test. Source data underlying Supplementary Figure 13a, 13c, and 13d are provided as a Source Data file.
