# Supplementary material for: Natural variation in a type-A response regulator confers maize chilling tolerance
Source: Nat Commun. 2021 Aug 5;12:4713. doi: 10.1038/s41467-021-25001-y (PMC8342596; doi:10.1038/s41467-021-25001-y)
Supplement: Supplementary file 9 — Description of Additional Supplementary Files [file 41467_2021_25001_MOESM9_ESM.docx]

**Description of Additional Supplementary Files**

File name: Supplementary data 1
Description: Cold-induced injured areas and haplotypes of inbred lines in maize.

File name: Supplementary data 2
Description: ZmRR1 regulated genes at permissive conditions (25°C).

File name: Supplementary data 3
Description: ZmRR1 regulated genes under cold conditions (4°C).

File name: Supplementary data 4
Description: ZmRR1-regulated genes in different clusters.

File name: Supplementary data 5
Description: ZmRR1-regulated cold responsive genes (803).

File name: Supplementary data 6
Description: List of the primers used in this study.
